# 1β,10α:4β,5α-Diep­oxy-7α*H*-germacran-6β-ol monohydrate

**DOI:** 10.1107/S1600536810047732

**Published:** 2010-11-20

**Authors:** Meng-Hui He, Quan Yang, Jie Sun

**Affiliations:** a School of Life Science, Foshan University, Foshan 528231, People’s Republic of China; b School of Chinese Medicine, Guangdong Pharmaceutical University, Guangzhou 510006, People’s Republic of China; c Shanghai Institute of Organic Chemistry, Chinese Academy of Sciences, Shanghai 20032, People’s Republic of China

## Abstract

In the title compound, C_15_H_26_O_3_·H_2_O, a sesquiterpenoid mol­ecule with a germacrene backbone that contains two epoxide groups and one hydroxyl group. Inter­molecular O—H⋯O hydrogen bonds between the ep­oxy groups and solvent water mol­ecules give rise to an infinite three-dimensional supra­molecular structure.

## Related literature

For the biosystematic and ecological evaluation of the title compound, see: Al Yousuf *et al.* (1999 ▶). For the isolation, see Li *et al.* (2009 ▶). For the determination of its absolute structure, see: Aguilar-Guadarrama & Rios (2004 ▶), Moodley *et al.* (2004 ▶). For related structures, see Takahashi *et al.* (1983 ▶), Barrero *et al.* (1999 ▶).
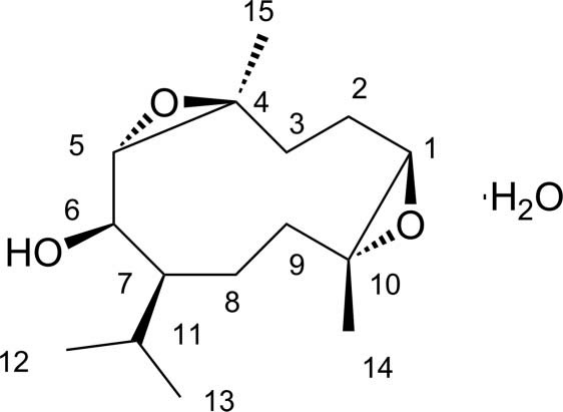


## Experimental

### 

#### Crystal data

C_15_H_26_O_3_·H_2_O
*M* *_r_* = 272.37
Orthorhombic, 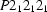

*a* = 8.087 (2) Å
*b* = 11.380 (3) Å
*c* = 16.943 (5) Å
*V* = 1559.2 (8) Å^3^
*Z* = 4
Mo *K*α radiation
μ = 0.08 mm^−1^
*T* = 293 K
0.31 × 0.13 × 0.07 mm

#### Data collection

Bruker SMART CCD area-detector diffractometer
Absorption correction: multi-scan (*SADABS*; Sheldrick, 2000 ▶) *T*_min_ = 0.757, *T*_max_ = 1.000
8291 measured reflections
1759 independent reflections
1281 reflections with *I* > 2σ(*I*)
*R*_int_ = 0.080

#### Refinement

*R*[*F* ^2^ > 2σ(*F* ^2^)] = 0.062
*wR*(*F* ^2^) = 0.148
*S* = 1.06
1759 reflections
186 parameters
H atoms treated by a mixture of independent and constrained refinement
Δρ_max_ = 0.16 e Å^−3^
Δρ_min_ = −0.15 e Å^−3^

### 

Data collection: *SMART* (Bruker, 2007 ▶); cell refinement: *SAINT* (Bruker, 2007 ▶); data reduction: *SAINT*; program(s) used to solve structure: *SHELXS97* (Sheldrick, 2008 ▶); program(s) used to refine structure: *SHELXL97* (Sheldrick, 2008 ▶); molecular graphics: *SHELXTL* (Sheldrick, 2008 ▶); software used to prepare material for publication: *SHELXTL*.

## Supplementary Material

Crystal structure: contains datablocks I, global. DOI: 10.1107/S1600536810047732/jj2065sup1.cif

Structure factors: contains datablocks I. DOI: 10.1107/S1600536810047732/jj2065Isup2.hkl

Additional supplementary materials:  crystallographic information; 3D view; checkCIF report

## Figures and Tables

**Table 1:** Hydrogen-bond geometry (Å, °)

*D*—H⋯*A*	*D*—H	H⋯*A*	*D*⋯*A*	*D*—H⋯*A*
O2—H3⋯O4^i^	0.82	2.03	2.819 (4)	160
O4—H4⋯O2^ii^	0.94 (6)	1.92 (6)	2.836 (5)	164 (5)
O4—H4*C*⋯O3	0.93 (4)	2.10 (5)	3.013 (4)	166 (4)

Symmetry codes: (i) 
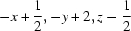
; (ii) 
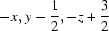
.

**Fig. 1.**
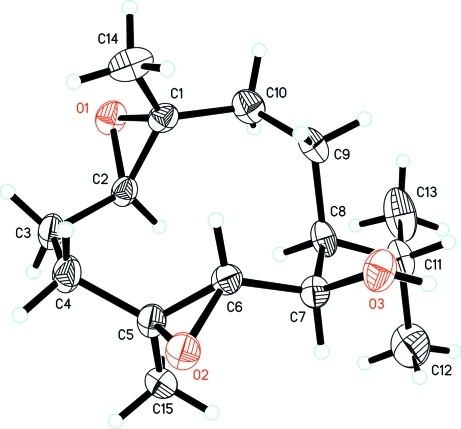
The molecular structure of the title compound, showing 30% displacement ellipsoids.

**Fig. 2.**
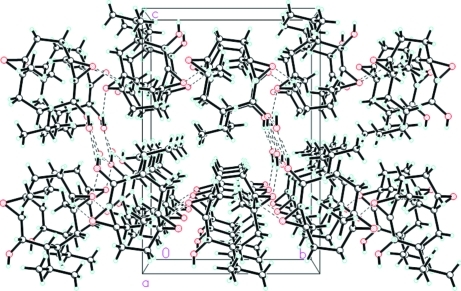
Packing diagram of the title compound viewed down the a axis. Dashed lines indicate O—H—O hydrogen bonds.

## supplementary crystallographic information

### Comment

Terpenoids are large naturally-occuring organic molecules derived from five-carbon isoprene units assembled and modified in numerous ways. A sesquiterpenoid is a 15-carbon modified multicyclic terpenoid hydrocarbon molecule that differs from one another in its functional groups.  The title compound, C_15_H_26_O_3_, H_2_O, is a sesquiterpenoid molecule with a germacrene backbone that contains two epoxide groups at the 1–10 and 4–5 ring positions and one alcohol group at the C6 position, respectively (Fig. 1). Crystal packing hss been stabilized as a result of a single water molecule that has crystallized in the unit cell giving rise to O—H···O intermolecular hydrogen bonds between the epoxy groups and the adjacent water molecules (Table 1).  This forms an infinite 3-D supramolecular structure (Fig. 2).

### Experimental

The title compound was synthesized by the method of Li *et al.* (2009). It was purified by repeated column chromatography over silica gel, ODS, and sephadex LH-20. It was then dissolved in a mixed solution of chloroform and methanol (*ca* 5:1). Colorless needle-like crystals were formed by slow evaporation of the solution in air.

### Refinement

All the H atoms were placed in idealized positions and constrained to ride on their parent atoms, with C—H (methine) distances of 0.98 Å, C—H (methylene) 0.97 Å, C—H (methyl) 0.96Å, and an O—H distance of 0.82 Å, with *U*_iso_(H) set at 1.2Ueq(C) and 1.5Ueq(O). A rotating group model was used for the OH group.

### Crystal data

**Table:**

C_15_H_26_O_3_·H_2_O	*F*(000) = 600
*M**_r_* = 272.37	*D*_x_ = 1.160 Mg m^−^^3^
Orthorhombic, *P*2_1_2_1_2_1_	Mo *K*α radiation, λ = 0.71073 Å
Hall symbol: P 2ac 2ab	Cell parameters from 1361 reflections
*a* = 8.087 (2) Å	θ = 4.8–39.9°
*b* = 11.380 (3) Å	µ = 0.08 mm^−^^1^
*c* = 16.943 (5) Å	*T* = 293 K
*V* = 1559.2 (8) Å^3^	Prismatic, colorless
*Z* = 4	0.31 × 0.13 × 0.07 mm

### Data collection

**Table:**

Bruker SMART CCD area-detector diffractometer	1759 independent reflections
Radiation source: fine-focus sealed tube	1281 reflections with *I* > 2σ(*I*)
graphite	*R*_int_ = 0.080
φ and ω scans	θ_max_ = 26.0°, θ_min_ = 2.2°
Absorption correction: multi-scan (*SADABS*; Sheldrick, 2000)	*h* = −9→9
*T*_min_ = 0.757, *T*_max_ = 1.000	*k* = −14→11
8291 measured reflections	*l* = −20→20

### Refinement

**Table:**

Refinement on *F*^2^	Secondary atom site location: difference Fourier map
Least-squares matrix: full	Hydrogen site location: inferred from neighbouring sites
*R*[*F*^2^ > 2σ(*F*^2^)] = 0.062	H atoms treated by a mixture of independent and constrained refinement
*wR*(*F*^2^) = 0.148	*w* = 1/[σ^2^(*F*_o_^2^) + (0.0727*P*)^2^] where *P* = (*F*_o_^2^ + 2*F*_c_^2^)/3
*S* = 1.06	(Δ/σ)_max_ = 0.005
1759 reflections	Δρ_max_ = 0.16 e Å^−^^3^
186 parameters	Δρ_min_ = −0.15 e Å^−^^3^
0 restraints	Extinction correction: *SHELXL97* (Sheldrick, 2008), Fc^*^=kFc[1+0.001xFc^2^λ^3^/sin(2θ)]^-1/4^
Primary atom site location: structure-invariant direct methods	Extinction coefficient: 0.010 (3)

### Special details

**Table:**

Geometry. All e.s.d.'s (except the e.s.d. in the dihedral angle between two l.s. planes) are estimated using the full covariance matrix. The cell e.s.d.'s are taken into account individually in the estimation of e.s.d.'s in distances, angles and torsion angles; correlations between e.s.d.'s in cell parameters are only used when they are defined by crystal symmetry. An approximate (isotropic) treatment of cell e.s.d.'s is used for estimating e.s.d.'s involving l.s. planes.
Refinement. Refinement of *F*^2^ against ALL reflections. The weighted *R*-factor *wR* and goodness of fit *S* are based on *F*^2^, conventional *R*-factors *R* are based on *F*, with *F* set to zero for negative *F*^2^. The threshold expression of *F*^2^ > σ(*F*^2^) is used only for calculating *R*-factors(gt) *etc*. and is not relevant to the choice of reflections for refinement. *R*-factors based on *F*^2^ are statistically about twice as large as those based on *F*, and *R*- factors based on ALL data will be even larger.

### Fractional atomic coordinates and isotropic or equivalent isotropic displacement parameters (Å^2^)

**Table:**

	*x*	*y*	*z*	*U*_iso_*/*U*_eq_	
O1	0.3976 (4)	1.2287 (2)	0.77838 (15)	0.0499 (8)	
O2	0.3551 (4)	1.2022 (2)	0.59240 (17)	0.0540 (9)	
H3	0.4081	1.2005	0.5512	0.081*	
O3	0.1549 (4)	0.7702 (2)	0.79520 (15)	0.0519 (8)	
O4	−0.0212 (5)	0.7428 (3)	0.9509 (2)	0.0686 (11)	
C1	0.2708 (6)	0.8691 (3)	0.7992 (2)	0.0402 (10)	
H1	0.3781	0.8546	0.7742	0.048*	
C2	0.2798 (6)	0.9355 (3)	0.8767 (2)	0.0505 (12)	
H2A	0.3750	0.9079	0.9063	0.061*	
H2B	0.1818	0.9180	0.9075	0.061*	
C3	0.2933 (6)	1.0692 (3)	0.8662 (2)	0.0527 (12)	
H3A	0.1844	1.1006	0.8545	0.063*	
H3B	0.3303	1.1038	0.9154	0.063*	
C4	0.4113 (6)	1.1048 (3)	0.8008 (2)	0.0420 (10)	
C5	0.3410 (5)	1.1380 (3)	0.7247 (2)	0.0396 (10)	
H5	0.2204	1.1304	0.7220	0.048*	
C6	0.4256 (5)	1.1207 (3)	0.64782 (19)	0.0402 (10)	
H6	0.5433	1.1386	0.6542	0.048*	
C7	0.4075 (5)	0.9917 (3)	0.6206 (2)	0.0432 (11)	
H7	0.4511	0.9430	0.6635	0.052*	
C8	0.2216 (5)	0.9560 (4)	0.6100 (2)	0.0482 (11)	
H8A	0.1534	1.0230	0.6240	0.058*	
H8B	0.2026	0.9393	0.5546	0.058*	
C9	0.1633 (7)	0.8502 (4)	0.6583 (2)	0.0534 (12)	
H9A	0.0630	0.8198	0.6344	0.064*	
H9B	0.2467	0.7892	0.6547	0.064*	
C10	0.1297 (5)	0.8741 (3)	0.7439 (2)	0.0427 (10)	
C11	0.5160 (6)	0.9655 (4)	0.5473 (2)	0.0539 (12)	
H11	0.4710	1.0109	0.5031	0.065*	
C12	0.6958 (6)	1.0003 (6)	0.5569 (3)	0.090 (2)	
H12A	0.7404	0.9632	0.6031	0.134*	
H12B	0.7036	1.0841	0.5625	0.134*	
H12C	0.7572	0.9758	0.5114	0.134*	
C13	0.5033 (9)	0.8344 (4)	0.5254 (3)	0.088 (2)	
H13A	0.3915	0.8162	0.5108	0.133*	
H13B	0.5348	0.7874	0.5700	0.133*	
H13C	0.5756	0.8179	0.4819	0.133*	
C14	−0.0276 (6)	0.9371 (4)	0.7621 (3)	0.0664 (14)	
H14A	−0.0342	1.0073	0.7309	0.100*	
H14B	−0.0299	0.9575	0.8171	0.100*	
H14C	−0.1198	0.8870	0.7502	0.100*	
C15	0.5860 (6)	1.0613 (4)	0.8082 (2)	0.0536 (12)	
H15A	0.6288	1.0823	0.8592	0.080*	
H15B	0.6530	1.0965	0.7679	0.080*	
H15C	0.5878	0.9774	0.8024	0.080*	
H4	−0.133 (8)	0.744 (5)	0.935 (3)	0.09 (2)*	
H4C	0.024 (6)	0.740 (4)	0.900 (3)	0.069 (15)*	

### Atomic displacement parameters (Å^2^)

**Table:**

	*U*^11^	*U*^22^	*U*^33^	*U*^12^	*U*^13^	*U*^23^
O1	0.065 (2)	0.0368 (15)	0.0480 (15)	−0.0061 (14)	0.0068 (15)	−0.0068 (13)
O2	0.061 (2)	0.0475 (18)	0.0531 (17)	0.0085 (16)	0.0069 (16)	0.0150 (14)
O3	0.065 (2)	0.0356 (15)	0.0556 (16)	−0.0072 (14)	0.0096 (16)	0.0006 (14)
O4	0.059 (3)	0.089 (3)	0.058 (2)	−0.023 (2)	0.0027 (19)	−0.021 (2)
C1	0.049 (3)	0.033 (2)	0.039 (2)	−0.0066 (19)	0.010 (2)	−0.0017 (18)
C2	0.068 (3)	0.046 (3)	0.038 (2)	−0.009 (2)	0.013 (2)	−0.0009 (19)
C3	0.068 (3)	0.049 (3)	0.042 (2)	−0.009 (2)	0.017 (2)	−0.011 (2)
C4	0.055 (3)	0.034 (2)	0.037 (2)	−0.003 (2)	0.006 (2)	−0.0017 (17)
C5	0.039 (2)	0.038 (2)	0.042 (2)	−0.0011 (18)	0.0045 (19)	−0.0060 (18)
C6	0.040 (3)	0.043 (2)	0.038 (2)	0.006 (2)	0.0017 (19)	0.0019 (18)
C7	0.058 (3)	0.042 (2)	0.0298 (17)	0.015 (2)	−0.0041 (19)	0.0029 (17)
C8	0.066 (3)	0.042 (2)	0.037 (2)	0.007 (2)	−0.014 (2)	−0.0026 (18)
C9	0.067 (3)	0.041 (3)	0.052 (3)	−0.002 (2)	−0.006 (2)	−0.002 (2)
C10	0.045 (3)	0.035 (2)	0.048 (2)	−0.0069 (19)	0.004 (2)	−0.0018 (18)
C11	0.071 (3)	0.058 (3)	0.033 (2)	0.020 (3)	−0.003 (2)	0.000 (2)
C12	0.058 (4)	0.138 (5)	0.073 (3)	0.026 (4)	0.018 (3)	−0.015 (4)
C13	0.147 (6)	0.067 (3)	0.051 (3)	0.042 (4)	0.007 (4)	−0.013 (2)
C14	0.046 (3)	0.056 (3)	0.097 (3)	−0.003 (2)	0.007 (3)	0.002 (3)
C15	0.055 (3)	0.060 (3)	0.046 (2)	−0.011 (2)	−0.006 (2)	−0.002 (2)

### Geometric parameters (Å, °)

**Table:**

O1—C5	1.450 (4)	C7—C8	1.568 (6)
O1—C4	1.464 (4)	C7—H7	0.9800
O2—C6	1.438 (4)	C8—C9	1.529 (5)
O2—H3	0.8200	C8—H8A	0.9700
O3—C1	1.467 (5)	C8—H8B	0.9700
O3—C10	1.481 (4)	C9—C10	1.501 (5)
O4—H4	0.94 (6)	C9—H9A	0.9700
O4—H4C	0.93 (4)	C9—H9B	0.9700
C1—C10	1.477 (6)	C10—C14	1.492 (6)
C1—C2	1.517 (5)	C11—C12	1.516 (7)
C1—H1	0.9800	C11—C13	1.541 (6)
C2—C3	1.536 (5)	C11—H11	0.9800
C2—H2A	0.9700	C12—H12A	0.9600
C2—H2B	0.9700	C12—H12B	0.9600
C3—C4	1.518 (5)	C12—H12C	0.9600
C3—H3A	0.9700	C13—H13A	0.9600
C3—H3B	0.9700	C13—H13B	0.9600
C4—C5	1.459 (5)	C13—H13C	0.9600
C4—C15	1.502 (6)	C14—H14A	0.9600
C5—C6	1.484 (5)	C14—H14B	0.9600
C5—H5	0.9800	C14—H14C	0.9600
C6—C7	1.545 (5)	C15—H15A	0.9600
C6—H6	0.9800	C15—H15B	0.9600
C7—C11	1.550 (6)	C15—H15C	0.9600
			
C5—O1—C4	60.1 (2)	C9—C8—C7	116.0 (3)
C6—O2—H3	109.5	C9—C8—H8A	108.3
C1—O3—C10	60.1 (2)	C7—C8—H8A	108.3
C1—O3—H4C	114.1 (12)	C9—C8—H8B	108.3
C10—O3—H4C	124.0 (13)	C7—C8—H8B	108.3
H4—O4—H4C	96 (4)	H8A—C8—H8B	107.4
O3—C1—C10	60.4 (2)	C10—C9—C8	115.4 (3)
O3—C1—C2	116.9 (3)	C10—C9—H9A	108.4
C10—C1—C2	124.6 (4)	C8—C9—H9A	108.4
O3—C1—H1	114.6	C10—C9—H9B	108.4
C10—C1—H1	114.6	C8—C9—H9B	108.4
C2—C1—H1	114.6	H9A—C9—H9B	107.5
C1—C2—C3	113.3 (3)	C1—C10—O3	59.4 (2)
C1—C2—H2A	108.9	C1—C10—C14	123.1 (3)
C3—C2—H2A	108.9	O3—C10—C14	112.3 (3)
C1—C2—H2B	108.9	C1—C10—C9	117.8 (4)
C3—C2—H2B	108.9	O3—C10—C9	113.4 (3)
H2A—C2—H2B	107.7	C14—C10—C9	116.2 (4)
C4—C3—C2	113.2 (3)	C12—C11—C13	110.1 (5)
C4—C3—H3A	108.9	C12—C11—C7	114.0 (4)
C2—C3—H3A	108.9	C13—C11—C7	110.0 (4)
C4—C3—H3B	108.9	C12—C11—H11	107.5
C2—C3—H3B	108.9	C13—C11—H11	107.5
H3A—C3—H3B	107.7	C7—C11—H11	107.5
C5—C4—O1	59.5 (2)	C11—C12—H12A	109.5
C5—C4—C15	121.8 (3)	C11—C12—H12B	109.5
O1—C4—C15	114.2 (4)	H12A—C12—H12B	109.5
C5—C4—C3	118.0 (4)	C11—C12—H12C	109.5
O1—C4—C3	113.5 (3)	H12A—C12—H12C	109.5
C15—C4—C3	116.2 (4)	H12B—C12—H12C	109.5
O1—C5—C4	60.5 (2)	C11—C13—H13A	109.5
O1—C5—C6	120.0 (3)	C11—C13—H13B	109.5
C4—C5—C6	124.1 (4)	H13A—C13—H13B	109.5
O1—C5—H5	114.0	C11—C13—H13C	109.5
C4—C5—H5	114.0	H13A—C13—H13C	109.5
C6—C5—H5	114.0	H13B—C13—H13C	109.5
O2—C6—C5	107.7 (3)	C10—C14—H14A	109.5
O2—C6—C7	112.4 (3)	C10—C14—H14B	109.5
C5—C6—C7	110.2 (3)	H14A—C14—H14B	109.5
O2—C6—H6	108.8	C10—C14—H14C	109.5
C5—C6—H6	108.8	H14A—C14—H14C	109.5
C7—C6—H6	108.8	H14B—C14—H14C	109.5
C6—C7—C11	111.6 (3)	C4—C15—H15A	109.5
C6—C7—C8	111.8 (3)	C4—C15—H15B	109.5
C11—C7—C8	113.7 (3)	H15A—C15—H15B	109.5
C6—C7—H7	106.4	C4—C15—H15C	109.5
C11—C7—H7	106.4	H15A—C15—H15C	109.5
C8—C7—H7	106.4	H15B—C15—H15C	109.5
			
H4C—O3—C1—C10	−116.7 (14)	O2—C6—C7—C8	−60.0 (4)
C10—O3—C1—C2	116.3 (4)	C5—C6—C7—C8	60.0 (4)
H4C—O3—C1—C2	−0.4 (14)	C6—C7—C8—C9	−123.0 (3)
O3—C1—C2—C3	−139.3 (4)	C11—C7—C8—C9	109.5 (4)
C10—C1—C2—C3	−68.2 (5)	C7—C8—C9—C10	77.7 (5)
C1—C2—C3—C4	−42.3 (6)	C2—C1—C10—O3	−104.0 (4)
C5—O1—C4—C15	113.9 (4)	O3—C1—C10—C14	98.1 (4)
C5—O1—C4—C3	−109.7 (4)	C2—C1—C10—C14	−5.8 (6)
C2—C3—C4—C5	100.5 (5)	O3—C1—C10—C9	−102.2 (4)
C2—C3—C4—O1	167.1 (4)	C2—C1—C10—C9	153.9 (4)
C2—C3—C4—C15	−57.4 (5)	H4C—O3—C10—C1	100.5 (15)
C4—O1—C5—C6	−114.7 (4)	C1—O3—C10—C14	−116.3 (4)
C15—C4—C5—O1	−101.2 (4)	H4C—O3—C10—C14	−15.8 (15)
C3—C4—C5—O1	102.2 (4)	C1—O3—C10—C9	109.5 (4)
O1—C4—C5—C6	108.0 (4)	H4C—O3—C10—C9	−150.0 (15)
C15—C4—C5—C6	6.8 (6)	C8—C9—C10—C1	−85.0 (5)
C3—C4—C5—C6	−149.8 (4)	C8—C9—C10—O3	−151.5 (4)
O1—C5—C6—O2	−84.7 (4)	C8—C9—C10—C14	76.1 (5)
C4—C5—C6—O2	−157.4 (3)	C6—C7—C11—C12	52.2 (5)
O1—C5—C6—C7	152.4 (3)	C8—C7—C11—C12	179.8 (4)
C4—C5—C6—C7	79.7 (5)	C6—C7—C11—C13	176.3 (4)
O2—C6—C7—C11	68.5 (4)	C8—C7—C11—C13	−56.2 (5)
C5—C6—C7—C11	−171.4 (3)		

### Hydrogen-bond geometry (Å, °)

**Table:**

*D*—H···*A*	*D*—H	H···*A*	*D*···*A*	*D*—H···*A*
O2—H3···O4^i^	0.82	2.03	2.819 (4)	160
O4—H4···O2^ii^	0.94 (6)	1.92 (6)	2.836 (5)	164 (5)
O4—H4C···O3	0.93 (4)	2.10 (5)	3.013 (4)	166 (4)

Symmetry codes: (i) −*x*+1/2, −*y*+2, *z*−1/2; (ii) −*x*, *y*−1/2, −*z*+3/2.

## References

[bb1] Aguilar-Guadarrama, A. B. & Rios, M. Y. (2004). *J. Nat. Prod.* **67**, 914–917. 10.1021/np030485f 15165166

[bb2] Al Yousuf, M. H., Bashir, A. K., Crabb, T. A., Blunden, G. & Yang, M.-H. (1999). *Biochem. System. Ecol.* **27**, 107–109.

[bb3] Barrero, A. F., Herrador, M. M., Quilez, J. F., Alvarez-Manzaneda, R., Portal, D., Gavin, J. A., Gravalos, D. G., Simmonds, M. S. J. & Blaney, W. M. (1999). *Phytochemistry*, **51**, 529–541. 10.1016/s0031-9422(99)00047-3 10389267

[bb4] Bruker (2007). *SMART* and *SAINT* Bruker AXS Inc., Madison, Wisconsin, USA.

[bb5] Li, S. M., Yang, X. W., Li, Y. L., Shen, Y. H., Feng, L., Wang, Y. H., Zeng, H. W., Liu, X. H., Zhang, C. S., Long, C. L. & Zhang, W. D. (2009). *Planta Med.* **75**, 1591–1596. 10.1055/s-0029-1185868 19579184

[bb6] Moodley, N., Mulholland, D. A. & Crouch, N. R. (2004). *J. Nat. Prod.* **67**, 918–920. 10.1021/np0204803 15165167

[bb7] Sheldrick, G. M. (2000). *SADABS* University of Göttingen, Germany.

[bb8] Sheldrick, G. M. (2008). *Acta Cryst.* A**64**, 112–122. 10.1107/S0108767307043930 18156677

[bb9] Takahashi, T., Nemoto, H., Tsuji, J. & Miura, I. (1983). *Tetrahedron Lett.* **24**, 3485–3488.

